# Tropifexor for nonalcoholic steatohepatitis: an adaptive, randomized, placebo-controlled phase 2a/b trial

**DOI:** 10.1038/s41591-022-02200-8

**Published:** 2023-02-16

**Authors:** Arun J. Sanyal, Patricia Lopez, Eric J. Lawitz, Kathryn J. Lucas, Juergen Loeffler, Won Kim, George B. B. Goh, Jee-Fu Huang, Carla Serra, Pietro Andreone, Yi-Cheng Chen, Stanley H. Hsia, Vlad Ratziu, Diego Aizenberg, Hiroshi Tobita, Aasim M. Sheikh, John M. Vierling, Yoon Jun Kim, Hideyuki Hyogo, Dean Tai, Zachary Goodman, Felicity Schaefer, Ian R. I. Carbarns, Sophie Lamle, Miljen Martic, Nikolai V. Naoumov, Clifford A. Brass

**Affiliations:** 1 Virginia Commonwealth University School of Medicine, Richmond, VA USA; 2 Novartis Pharma AG, Basel, Switzerland; 3 Texas Liver Institute, University of Texas Health, San Antonio, TX USA; 4 Diabetes and Endocrinology Consultants, Morehead City, NC USA; 5 Division of Gastroenterology and Hepatology, Department of Internal Medicine, Seoul National University College of Medicine, Seoul Metropolitan Government Boramae Medical Center, Seoul, Republic of Korea; 6 Department of Gastroenterology and Hepatology, Singapore General Hospital, Singapore, Singapore; 7 Hepatitis Centre and Hepatobiliary Division, Department of Internal Medicine, Kaohsiung Medical University Hospital, Kaohsiung City, Taiwan; 8 Diagnostic and Therapeutic Interventional Ultrasound Unit, IRCCS, Azienda Ospedaliero-Universitaria, Bologna, Italy; 9 University of Modena and Reggio Emilia, Modena, Italy; 10 Azienda Ospedaliero-Universitaria di Modena, Modena, Italy; 11 Department of Gastroenterology and Hepatology, Chang Gung Memorial Hospital, Chang Gung University College of Medicine, Taoyuan, Taiwan; 12 National Research Institute, Los Angeles, CA USA; 13 Sorbonne Université, Assistance Publique-Hôpitaux de Paris, Hôpital Pitié Salpêtrière, Institute of Cardiometabolism and Nutrition (ICAN), Paris, France; 14 Centro Medico Viamonte, Buenos Aires, Argentina; 15 Shimane University Hospital, Izumo, Japan; 16 Gastrointestinal Specialists of Georgia, Marietta, GA USA; 17 Advanced Liver Therapies, Baylor College of Medicine, Houston, TX USA; 18 Seoul National University College of Medicine and Liver Research Institute, Seoul, Korea; 19 JA Hiroshima General Hospital, Hiroshima, Japan; 20 Life Care Clinic Hiroshima, Hiroshima, Japan; 21 HistoIndex Pte. Ltd, Singapore, Singapore; 22 Inova Fairfax Hospital, Falls Church, VA USA; 23 Novartis Pharmaceutical Corporation, East Hanover, NJ USA

**Keywords:** Drug development, Medical research

## Abstract

The multimodal activities of farnesoid X receptor (FXR) agonists make this class an attractive option to treat nonalcoholic steatohepatitis. The safety and efficacy of tropifexor, an FXR agonist, in a randomized, multicenter, double-blind, three-part adaptive design, phase 2 study, in patients with nonalcoholic steatohepatitis were therefore assessed. In Parts A + B, 198 patients were randomized to receive tropifexor (10–90 μg) or placebo for 12 weeks. In Part C, 152 patients were randomized to receive tropifexor 140 µg, tropifexor 200 µg or placebo (1:1:1) for 48 weeks. The primary endpoints were safety and tolerability to end-of-study, and dose response on alanine aminotransferase (ALT), aspartate aminotransferase (AST) and hepatic fat fraction (HFF) at week 12. Pruritus was the most common adverse event in all groups, with a higher frequency in the 140- and 200-µg tropifexor groups. Decreases from baseline in ALT and HFF were greater with tropifexor versus placebo at week 12, with a relative decrease in least squares mean from baseline observed with all tropifexor doses for ALT (tropifexor 10–90-μg dose groups ranged from −10.7 to −16.5 U l^−1^ versus placebo (−7.8 U l^−1^) and tropifexor 140- and 200-μg groups were −18.0 U l^−1^ and −23.0 U l^−1^, respectively, versus placebo (−8.3 U l^−1^)) and % HFF (tropifexor 10–90-μg dose groups ranged from −7.48% to −15.04% versus placebo (−6.19%) and tropifexor 140- and 200-μg groups were −19.07% and −39.41%, respectively, versus placebo (−10.77%)). Decreases in ALT and HFF were sustained up to week 48; however, similar trends in AST with tropifexor at week 12 were not observed. As with other FXR agonists, dose-related pruritus was frequently observed. Clinicaltrials.gov registration: NCT02855164

## Abstract (web summary)

The FLIGHT-FXR trial, testing farnesoid X receptor agonist tropifexor in nonalcoholic steatohepatitis, demonstrated sustained decreases in alanine aminotransferase levels and hepatic fat fraction, but not aspartate aminotransferase, with dose-dependent pruritus being the most common adverse event.

## Main

Nonalcoholic fatty liver disease (NAFLD) is one of the most common chronic liver diseases worldwide^1,2^. Nonalcoholic steatohepatitis (NASH), the progressive subtype of NAFLD, is characterized by the presence of steatosis, lobular inflammation and hepatocyte ballooning, with or without hepatic fibrosis^3,4^. In the United States, the estimated prevalence of NASH in patients with NAFLD is ~25% (ref. ^2^), while in the general population it is estimated at 1.5–6.45% (ref. ^1^). NASH is a risk factor for progression toward advanced fibrosis and cirrhosis with subsequent risks for hepatocellular carcinoma, portal hypertension, end-stage liver disease and death^5–8^. Evidence from the United States suggests that NAFLD/NASH is the second leading cause for liver transplantation^9^ and a risk factor for all-cause mortality^10^.

Development of NASH treatments presents a myriad of unique challenges due to the complex pathophysiology of the disease, and there are currently no approved pharmacological therapies^3,5,6,11^. Lifestyle intervention with the goal of ≥7% weight loss has been associated with histologic improvement^3,4,12^. Given that patients are frequently unable to achieve and sustain such weight loss levels, there is a major unmet need for pharmacological treatments^5,12^.

The FXR is a nuclear receptor that is physiologically activated by bile acids and is expressed at high levels in the liver and intestine^13^. A key regulator of bile acid production, conjugation and elimination^13^, FXR also modulates hepatic triglyceride and glucose metabolism^14^. Low levels of hepatic FXR have been reported in patients with NAFLD and are inversely associated with disease severity, suggesting a role in the pathogenesis of the disease^15^. A role for FXR agonism for the treatment of NASH has been demonstrated in clinical trials with obeticholic acid, a synthetically modified variant of the natural bile acid chenodeoxycholic acid^16,17^; several non-bile-acid FXR agonists are also in clinical development^18^.

Tropifexor is a selective, non-bile-acid FXR agonist that has shown high potency of target engagement and treatment efficacy in animal models of NASH^19,20^. Furthermore, in a first-in-human study in healthy volunteers, tropifexor at single doses up to 3,000 μg was safe and well-tolerated, with a pharmacokinetic (PK) profile suitable for once-daily dosing^21^. The non-bile-acid structure of tropifexor provides unique features, including an absence of enterohepatic circulation and a low potential for off-target activation of the cell surface G protein-coupled bile acid receptor 1, which differentiate it from bile-acid-based FXR agonists and alter the therapeutic index^19,20,22,23^.

The results of FLIGHT-FXR, a phase 2 randomized, multicenter, double-blind, three-part study with adaptive design, are presented here. Safety, tolerability and efficacy of multiple, once-daily doses of tropifexor in patients with NASH versus placebo for 12 (Parts A and B) or 48 weeks (Part C) were examined. As per a planned analysis, data from Parts A and B have been pooled and expressed as ‘Parts A + B’ in this publication.

## Results

### Patient disposition and baseline demographics

**Fig. 1:**
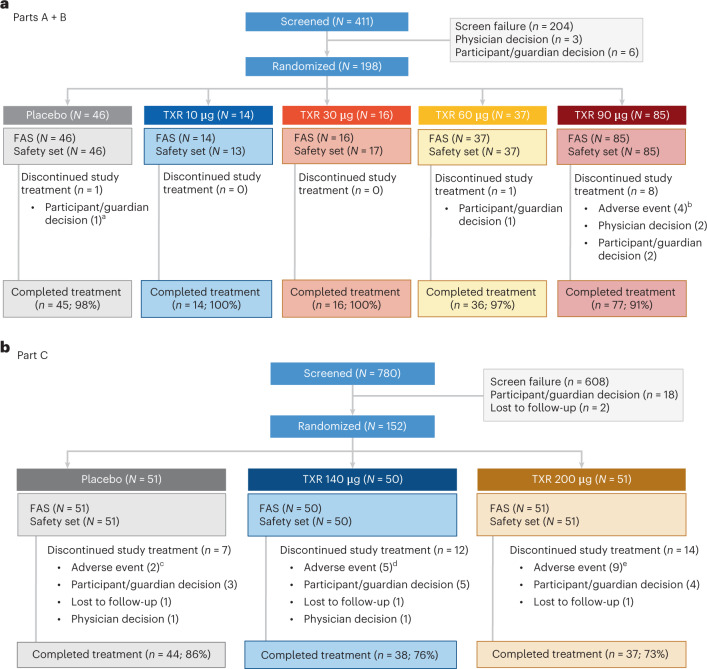
Patient disposition. **a**, Parts A + B. **b**, Part C. The figure reports the primary reason for discontinuation. ^a^1 patient discontinued due to AE (Table 2); however, the AE was not the ‘primary’ reason for discontinuation. ^b^5 patients discontinued due to AEs (Table 2); however, only 4 patients reported AEs as the primary reason for discontinuation (CK increased (1), constipation (1), pruritus (1), T2DM (1)). ^c^AST increased (1), back pain (1). ^d^Pruritus (3), AST increased (1), drug eruption (1). ^e^9 patients had a total of 12 AEs leading to discontinuation: pruritus (4), abdominal pain (2), AST increased (1), blood ALP increased (1), breast pain (1), diarrhea (1), increased liver stiffness, (1) oral paresthesia (1). CK, creatine phosphokinase; FAS, full analysis set; TXR, tropifexor.

**Extended Data Fig. 1:**
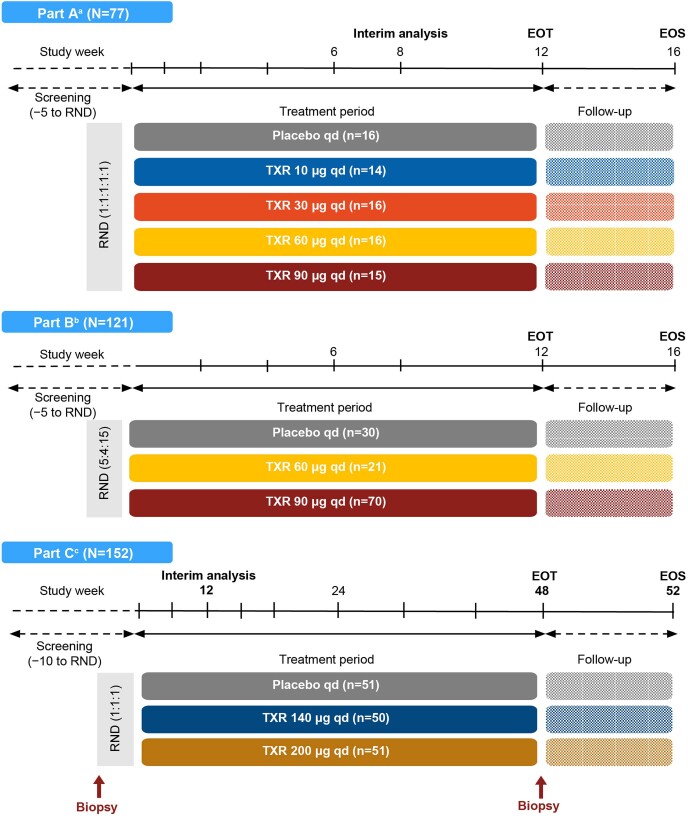
Study design. ^a^When ≥90% of the patients in Part A completed 8 weeks of treatment, an interim analysis was performed to allow for the DMC to recommend dose selection for Part B. ^b^Randomization to Part B commenced after the DMC recommendation on dose selection. ^c^Randomization into Part C began after completion of Part B randomization. DMC, data monitoring committee; EOS, end-of-study; EOT, end-of-treatment; qd, once daily; RND, randomization; TXR, tropifexor.

Of the 411 patients screened, 198 (48.2%) were randomized in Parts A and B. In Part A, 77 patients were randomized to receive placebo (*n* = 16) or tropifexor 10 μg (*n* = 14), 30 μg (*n* = 16), 60 μg (*n* = 16) or 90 μg (*n* = 15). In Part B, 121 patients were randomized to receive placebo (*n* = 30), tropifexor 60 μg (*n* = 21) or tropifexor 90 μg (*n* = 70). Study design and number of patients per treatment group are shown in Extended Data Fig. 1. All patients in the tropifexor 10- and 30-μg groups completed treatment. Treatment discontinuation rates were higher in the tropifexor 90-μg (8 of 85; 9%) group versus placebo (1 of 46; 2%) and tropifexor 60-μg (1 of 37; 3%) groups. The primary reason for treatment discontinuation was participant decision in the placebo and tropifexor 60-μg groups and adverse events (AEs; *n* = 4), participant/guardian decision (*n* = 2) and physician decision (*n* = 2) in the tropifexor 90-μg group (Fig. 1a). A total of 780 patients were screened in Part C. Of these, 152 (19.5%) were randomized to receive placebo (*n* = 51), tropifexor 140 µg (*n* = 50) or tropifexor 200 µg (*n* = 51). The screen failure rate in Part C was greater than that in Parts A and B due to the additional requirement in Part C for adequate liver biopsy samples for evaluation by the central pathologist to confirm histologic evidence of NASH with fibrosis stage 2 or 3. No Part C patients were included based on phenotypic diagnosis of NASH alone. Treatment discontinuation rates were higher in the tropifexor 140-μg (24%) and 200-μg (27%) groups versus placebo (14%). AEs were the most common reason for treatment discontinuation in the tropifexor 140-μg (*n* = 5) and 200-μg (*n* = 9) groups compared with placebo (*n* = 2), followed by participant/guardian decision (tropifexor 140 μg: *n* = 5; tropifexor 200 μg: *n* = 4; placebo: *n* = 3; Fig. 1b). No patient in any study part discontinued treatment due to noncompliance with study treatment or protocol deviation.

Demographics and baseline characteristics were generally balanced between groups in all study parts (Table 1). In Parts A + B, the majority of patients were female (53%) and white (62%). Other reported races were: Black (1%), Asian (35%), Pacific Islander (1%), unknown (1%) and other (1%). Of 198 patients, 78 (39%) had NASH confirmed by historical biopsy and in 120 (61%) the diagnosis of NASH was not confirmed by historical biopsy. Similar to Parts A + B, the majority of patients in Part C were female (64%) and White (74%). Other reported races were: Black (1%), Asian (18%), Pacific Islander (1%) and other (6%). Approximately 59% of patients in Part C had stage 3 fibrosis at baseline. The mean HFF at baseline was 20.2% in the placebo group and 18.1% in both the tropifexor 140- and 200-µg groups. The mean NAFLD activity score (NAS) total was 6 in the placebo group and both tropifexor groups before study treatment.

**Table 1:** Demographics and baseline characteristics (FAS)

		Pooled Parts A + B					Part C		
		Placebo	Tropifexor				Placebo	Tropifexor	
Characteristics (data are mean (s.d.) unless specified)			10 μg	30 μg	60 μg	90 μg		140 μg	200 μg
		*N* = 46	*N* = 14	*N* = 16	*N* = 37	*N* = 85	*N* = 51	*N* = 50	*N* = 51
Age (yr)		51 (12.3)	48 (11.7)	49 (14.4)	50 (12.5)	51 (13.4)	54 (11.0)	56 (11.4)	55 (10.8)
Female, *n* (%)		21 (46)	9 (64)	7 (44)	20 (54)	47 (55)	32 (63)	36 (72)	29 (57)
Race, *n* (%)									
White		25 (54)	12 (86)	11 (69)	24 (65)	50 (59)	38 (75)	37 (74)	38 (75)
Asian		20 (43)	2 (14)	5 (31)	12 (32)	31 (36)	8 (16)	10 (20)	10 (20)
Others^a^		1 (2)	0	0	1 (3)	4 (5)	5 (10)	3 (6)	3 (6)
Weight (kg)		91 (20.2)	94 (17.4)^b^	93 (15.6)	94 (20.6)	88 (19.1)	95 (24.5)	92 (17.2)^c^	96 (18.1)
BMI (kg m^−2^)		32 (6.7)	33 (3.8)	33 (5.4)	33 (5.5)	32 (5.6)	35 (7.2)	34 (5.6)	34 (5.5)
Diabetes status (Yes),^d,e^ *n* (%)		31 (67)	11 (79)	11 (69)	29 (78)	64 (75)	40 (78)	39 (78)	43 (84)
ALT (U l^−1^)		80 (36.8)	72 (21.9)	83 (34.9)	81 (44.6)	83 (39.2)	75 (38.7)	62 (28.6)	75 (46.2)
AST (U l^−1^)		55 (28.9)	54 (18.5)	54 (25.6)	59 (37.8)	58 (26.8)	59 (25.4)	51 (18.7)	60 (36.6)
GGT (U l^−1^)		65 (43.0)	72 (69.1)	68 (37.2)	61 (43.6)	86 (77.1)	69 (42.2)	63 (45.9)	71 (58.7)
HFF (%)		20 (6.4)	22 (8.6)	19 (5.8)	19 (5.3)	21 (7.2)	20 (7.8)	18 (6.9)	18 (6.3)
LDL-C (mg dl^−1^)		118 (26.2)	106 (24.1)	107 (25.8)	118 (35.1)	124 (38.6)	121 (45.9)	118 (34.3)	101 (30.8)
HDL-C (mg dl^−1^)	54 (13.8)		52 (13.4)	48 (8.4)	50 (12.4)	51 (15.1)	47 (11.9)	50 (13.3)	50 (15.0)
FIB-4	1.5 (1.09)		1.5 (0.82)	1.3 (0.92)	1.5 (0.71)	1.5 (0.84)	1.6 (0.69)	1.8 (1.22)	1.9 (1.56)
Diagnosis of NASH confirmed by historic biopsy results, *n* (%)	18 (39)		6 (43)	8 (50)	12 (32)	34 (40)	51 (100)	50 (100)	51 (100)
Fibrosis stage (NASH CRN), *n* (%)									
2	–		–	–	–	–	22 (43)	20 (40)	21 (41)
3	–		–	–	–	–	29 (57)	30 (60)	30 (59)
NAS-total, mean (s.d.)	–		–	–	–	–	6 (0.7)	6 (0.5)	6 (0.7)
Ongoing protocol solicited medical history, *n* (%)^f^									
Hepatic steatosis	18 (39)		3 (21)	9 (56)	17 (46)	42 (49)	31 (61)	31 (62)	34 (67)
Hypertension	26 (57)		8 (57)	7 (44)	20 (54)	47 (55)	34 (67)	32 (64)	35 (69)
NASH	45 (98)		14 (100)	15 (94)	37 (100)	84 (99)	50 (98)	50 (100)	49 (96)
NAFLD	18 (39)		9 (64)	7 (44)	21 (57)	39 (46)	35 (69)	30 (60)	36 (71)
Steatohepatitis	16 (35)		3 (21)	7 (44)	13 (35)	35 (41)	26 (51)	22 (44)	28 (55)
Type 2 diabetes	24 (52)		9 (64)	9 (56)	22 (59)	56 (66)	30 (59)	28 (56)	30 (59)

^a^Others include Black, Pacific Islander, Unknown and Other for Parts A + B, and Black, Pacific Islander and Other for Part C.

^b^*n* = 13.

^c^*n* = 49.

^d^Defined based on medical history of type 1 or type 2 diabetes or fasting glucose >100 mg dl^−1^ (>5.6 mmol l^−1^), 1 patient was reported to have type 1 diabetes in Part C (placebo arm).

^e^In terms of concomitant diabetic medications, there were 8 patients treated with glucagon-like peptide 1 (GLP-1) agonists, 18 with sodium glucose cotransporter 2 (SGLT-2) inhibitors and 5 with pioglitazone in Parts A + B. In Part C, there were 19 patients treated with GLP-1 agonists, 23 with SGLT-2 inhibitors and 7 with pioglitazone.

^f^Terms with >50% occurrence in any study group.

APRI, AST Platelet Ratio Index; FAS, full analysis set; FIB-4, Fibrosis-4; *N*, patient number (treatment group); *n*, number of patients with reported values.

### Safety and tolerability

In Parts A + B, the overall rates of AEs were comparable between the placebo (67%) and tropifexor 30–90-μg (65–72%) groups, with a lower incidence in the tropifexor 10-µg (38%) group. Serious AEs (SAEs; *n* = 4) were only reported in the tropifexor 90-µg group. Pruritus, fatigue and nasopharyngitis were the most common AEs reported in the placebo and tropifexor groups, with no consistent elevation of pruritus at these doses (Table 2).

**Table 2:** Overall safety and tolerability (safety analysis set)

	Pooled Parts A + B (week 12)					Part C (week 48)		
	Placebo	Tropifexor				Placebo	Tropifexor	
		10 μg^a^	30 μg^a^	60 μg	90 μg		140 μg	200 μg
Incidence, *n* (%)	*N* = 46	*N* = 13	*N* = 17	*N* = 37	*N* = 85	*N* = 51	*N* = 50	*N* = 51
Number of participants with at least one AE	31 (67)	5 (38)	11 (65)	24 (65)	61 (72)	46 (90)	49 (98)	49 (96)
Number of participants with at least one SAE	0	0	0	0	4 (5)^b^	6 (12)^c^	5 (10)^d^	3 (6)^e^
AEs leading to dose reduction/discontinuation	1 (2)	0	0	0	8 (9)	3 (6)	9 (18)	19 (37)
AEs leading to discontinuation	1 (2)	0	0	0	5 (6)	2 (4)	5 (10)	9 (18)
SAEs leading to discontinuation	0	0	0	0	2 (2)	0	0	0
Death	0	0	0	0	0	0	0	0
**AEs of interest**^f^								
Pruritus	4 (9)	0	0	5 (14)	7 (8)	11 (22)	26 (52)	35 (69)
Grade 1	4 (9)	0	0	5 (14)	5 (6)	10 (20)	16 (32)	20 (39)
Grade 2	0	0	0	0	1 (1)	1 (2)	8 (16)	11 (22)
Grade 3	0	0	0	0	1 (1)	0	2 (4)	4 (8)
Nausea	4 (9)	0	0	1 (3)	4 (5)	7 (14)	6 (12)	10 (20)
Influenza	1 (2)	0	0	0	9 (11)	3 (6)	1 (2)	3 (6)
Upper respiratory tract infection	2 (4)	0	0	2 (5)	8 (9)	8 (16)	9 (18)	3 (6)
Diarrhea	1 (2)	0	1 (6)	1 (3)	4 (5)	5 (10)	3 (6)	7 (14)
Nasopharyngitis	6 (13)	0	0	2 (5)	6 (7)	4 (8)	6 (12)	5 (10)
Fatigue	5 (11)	0	3 (18)	1 (3)	5 (6)	4 (8)	7 (14)	3 (6)

^a^1 patient assigned to tropifexor 10-μg group erroneously received tropifexor 30 μg for some portion (~10 d) of the initial 4 weeks of treatment. This patient was included under tropifexor 10-μg group for efficacy analyses and under tropifexor 30-μg group for safety analyses.

^b^Hematochezia (*n* = 1), blood creatine phosphokinase increased (*n* = 1), arthralgia (*n* = 1) and renal impairment (*n* = 1). Tropifexor was discontinued for the case of blood creatine phosphokinase increase (participant involved in high-intensity sports activity which provided a plausible explanation for the event; however, a relationship between study drug and event was not excluded by the investigator) and for the case of renal impairment (reviewed by a renal physician who concluded the event to be due to diabetic nephropathy and not related to study drug).

^c^Cholecystitis acute (*n* = 1), device dislocation (*n* = 1), gastroenteritis (*n* = 1), multiple injuries (*n* = 1), malignant melanoma (*n* = 1), transient ischemic attack (*n* = 1) and hemothorax (*n* = 1). Study medication interrupted for the case of cholecystitis acute and for the case of transient ischemic attack.

^d^Angina pectoris and tachycardia (*n* = 1), noncardiac chest pain (*n* = 1), animal bite (*n* = 1), synovial cyst (*n* = 1), trigger finger (*n* = 1) and endometrial thickening (*n* = 1). Tropifexor was briefly interrupted for the case of animal bite.

^e^Angina pectoris (*n* = 1), hyperglycemia (*n* = 1), nephrolithiasis (*n* = 1). No dose interruptions or discontinuations reported.

^f^AEs most frequently reported in Parts A + B or Part C.

*n*, number of patients with reported values; *N*, patient number (treatment group).

In Part C, overall AEs were more frequent in the tropifexor 140- and 200-µg groups (98% and 96%, respectively), versus placebo group (90%); however, the incidence of SAEs was numerically lower with tropifexor 140 and 200 µg (10% and 6%, respectively) versus placebo (12%). Although treatment discontinuation resulted from AEs in some cases (Table 2), none of the AEs that led to treatment discontinuation were serious. Pruritus was the most common AE reported in the placebo (22%), tropifexor 140-μg (52%) and tropifexor 200-μg (69%) groups. Although none of the pruritus events were serious, the severity of events appeared to increase with tropifexor dose. Dose reduction or discontinuation of study drug due to pruritus was higher in patients receiving tropifexor 140 μg (12% (*n* = 6), of which discontinuations were 6% (*n* = 3)) and tropifexor 200 μg (25% (*n* = 13), of which discontinuations were 8% (*n* = 4)) versus placebo (0%, no dose reductions or discontinuations). There was no evidence of drug-induced liver injury during the study, with no participants meeting Hy’s law criteria. One cholecystitis event was noted in each of the placebo and tropifexor 140-μg groups.

### ALT, AST and HFF

**Fig. 2:**
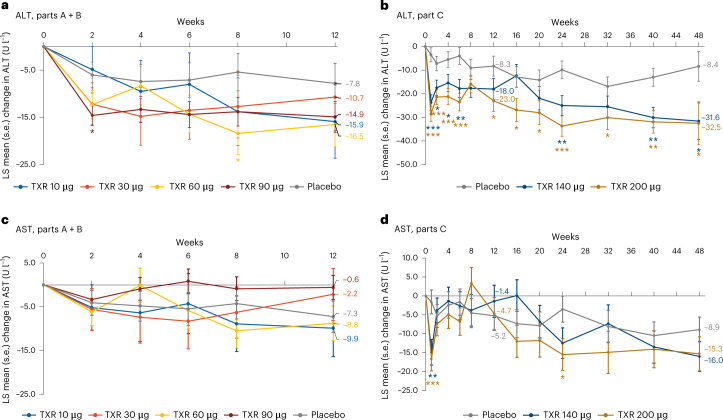
Change in ALT and AST from baseline up to end-of-treatment. **a**, ALT Parts A + B. **b**, ALT Part C. **c**, AST Parts A + B. **d**, AST Part C. *P < 0.05, **P < 0.01, ***P < 0.001 versus placebo. Data are presented as LS mean change (s.e.) with two-sided unadjusted *P* values from repeated measures ANCOVA.

At week 12, the least squares (LS) mean decreases in ALT from baseline in the tropifexor 10–90-μg dose groups ranged from −10.7 to −16.5 U l^−1^ and were greater than in the placebo group (−7.8 U l^−1^) (Fig. 2a). Greater LS mean decreases in ALT were noted with higher tropifexor doses versus placebo and were sustained up to week 48 (tropifexor 140 μg, −31.6 U l^−1^; tropifexor 200 μg, −32.5; versus placebo, −8.4 U l^−1^; Fig. 2b). *P* values < 0.05 are indicated by asterisks in Fig. 2 but cannot be formally claimed to be statistically significant because there was no adjustment for multiplicity.

The LS mean decrease in aspartate aminotransferase (AST) from baseline to week 12 was −7.3 U l^−1^ in the placebo group and ranged from −0.6 to −9.9 U l^−1^ in the tropifexor 10–90-μg groups (Fig. 2c). At week 48, the LS mean decreases in AST from baseline were −8.9 U l^−1^ in the placebo group and −16.0 U l^−1^ and −15.3 U l^−1^ in the tropifexor 140- and 200-μg groups, respectively (Fig. 2d).

**Fig. 3:**
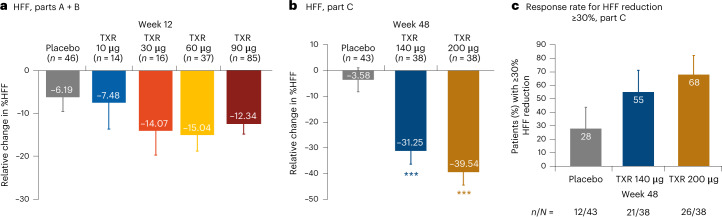
Change from baseline in HFF. **a**, HFF Parts A + B. **b**, HFF Part C**. c**, Response rate for HFF reduction ≥30% Part C. ***P < 0.001 versus placebo. Data are presented as LS mean change (s.e.) with two-sided unadjusted *P* values from repeated measures ANCOVA (HFF, Part C) or ANCOVA (HFF, Parts A + B). Response rate was defined as % patients achieving HFF reduction of ≥30% and is presented as response rate with 95% CI.

The relative decrease from baseline in LS mean % HFF at week 12 ranged from −7.48% to −15.04% in the tropifexor 10–90-μg dose groups and was consistently greater versus placebo (−6.19%) (Fig. 3a). A dose-dependent relative decrease in LS mean % HFF at week 12 was observed with tropifexor 140 (−19.07%; *P* = 0.124) and 200 μg (−39.41%; *P* < 0.001) compared with placebo (−10.77%) and continued to decrease further at week 48 (−31.25% and −39.54% versus −3.58%; *P* < 0.001 for both comparisons; Fig. 3b). The proportion of patients achieving relative HFF reduction by ≥30% at week 48 was 28% in the placebo group and 55% and 68% in the tropifexor 140- and 200-µg groups, respectively (Fig. 3c). Pairwise differences with 95% confidence interval (CI) for ALT, AST and relative change in HFF are summarized in Extended Data Table 1.

### Lipids

**Extended Data Fig. 2:**
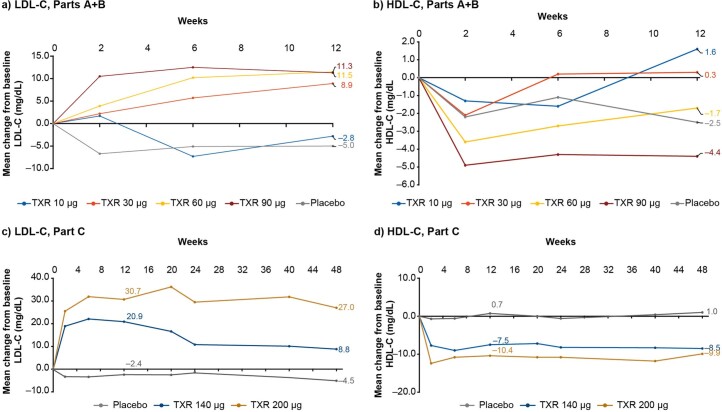
Mean change from baseline in LDL-C and HDL-C. a, LDL-C, Parts A+B. b, HDL-C, Parts A+B. c, LDL-C, Part C. d, HDL-C, Part C. HDL-C, high-density lipoprotein cholesterol; LDL-C, low-density lipoprotein cholesterol; TXR, tropifexor.

Tropifexor treatment was associated with an overall trend of increase in low-density lipoprotein cholesterol (LDL-C) and decrease in high-density lipoprotein cholesterol (HDL-C) levels (Extended Data Fig. 2); however, few patients were initiated on lipid-lowering drugs during the study (placebo, *n* = 1; tropifexor 140 μg, *n* = 0; tropifexor 200 μg, *n* = 2) and changes in LDL-C and HDL-C levels in the tropifexor groups stabilized after week 12. At week 12, the mean change from baseline in LDL-C levels (mg dl^−1^) for tropifexor 10–90-μg dose groups ranged from −2.82 to +11.48, versus placebo (−4.99), and tropifexor 140- and 200-μg doses were +20.94 and +30.75, respectively, versus placebo (−2.37). At week 48, the values were −4.52 in the placebo group and +8.8 and +26.96 in the tropifexor 140- and 200-µg groups, respectively. The mean change from baseline in HDL-C levels (mg dl^−1^) at week 12 for tropifexor 10–90-μg dose groups ranged from +1.58 to −4.42, versus placebo (−2.46), and tropifexor 140- and 200-μg doses were −7.52 and −10.46, respectively, versus placebo (+0.74). At week 48, the values were +1.08 in the placebo group and −8.55 and −9.88 in the tropifexor 140- and 200-µg groups. None of the patients discontinued treatment due to dyslipidemia. No deaths were reported during the study.

### Target engagement

FXR target engagement was confirmed at week 6 by dose-dependent increases in fibroblast growth factor 19 (FGF19) levels and decreases in 7α-Hydroxy-4-cholesten-3-one (C4) levels with tropifexor 60–200-µg doses compared with placebo. The LS geometric mean ratio of FGF19 postdose (at 4 h) to predose and the LS geometric mean ratio of C4 at end-of-treatment to baseline have been summarized in Extended Data Table 2. The treatment-related decrement in C4 was in effect all day as there were no pre- and postdose differences in C4 levels at week 6.

### PKs

In Parts A + B, at day 7, an 8.6-fold increase in the mean predose concentration of tropifexor in plasma was observed for a 9.0-fold increase in dose. Similarly, for 2 h postdose mean concentrations there was an 11.5-fold increase in mean concentration for a 9.0-fold increase in dose. Mean predose concentrations showed minimal fluctuation over the study duration. In Part C, mean predose concentrations showed minimal fluctuation over the study duration. Mean postdose tropifexor concentrations were only slightly increased relative to mean predose concentrations (data not shown).

### Anthropometrics

**Extended Data Fig. 3:**
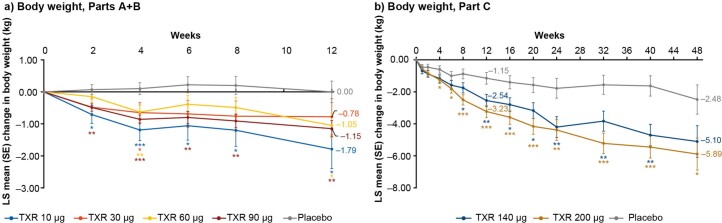
Change from baseline in body weight. a, Body weight Parts A+B. b, Body weight Part C. *P<0.05, **P<0.01, ***P<0.001 versus placebo. Data are presented as LS mean change (SE) with two-sided unadjusted P values from repeated measures ANCOVA. ANCOVA, analysis of covariance; LS, least squares; SE, standard error; TXR, tropifexor.

Although no change in body weight was noted in the placebo group at week 12, the LS mean decrease was greater in the tropifexor 10- (−1.79 kg), 60- (−1.05 kg) and 90-μg (−1.15 kg) groups versus placebo (0.00 kg) (Extended Data Fig. 3a). A dose-dependent reduction in body weight was observed with tropifexor 140 and 200 μg compared with placebo. At week 48, the LS mean decrease in body weight was greater in the tropifexor 140- (−5.10 kg) and 200-μg (−5.89 kg) groups versus placebo (−2.48 kg) (Extended Data Fig. 3b). Similar trends in body mass index (BMI) were observed (data not shown). No notable change from baseline to week 12 in waist-to-hip ratio was observed in any tropifexor dose group compared with placebo. Pairwise differences with 95% CIs for body weight are summarized in Extended Data Table 3.

### Gamma-glutamyl transferase, alkaline phosphatase and markers of liver fibrosis

**Extended Data Fig. 4:**
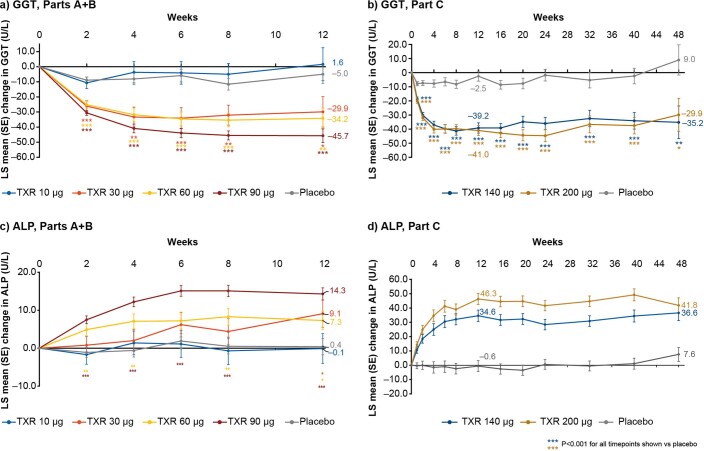
Changes in GGT and ALP from baseline to end-of-treatment. a, GGT, Parts A+B. b, GGT, Part C. c, ALP, Parts A+B. d, ALP, Part C. *P<0.05, **P<0.01, ***P<0.001 versus placebo. Data are presented as LS mean change (SE) with two-sided unadjusted P values from repeated measures ANCOVA. ALP, alkaline phosphatase; ANCOVA, analysis of covariance; GGT, gamma-glutamyl transferase; LS, least squares; SE, standard error; TXR, tropifexor.

A marked reduction in gamma-glutamyl transferase (GGT) levels was evident from week 2 with tropifexor 30–90-μg doses and from week 1 with tropifexor 140- and 200-μg doses, which was sustained to end-of-treatment. The LS mean decrease in GGT from baseline at week 12 was higher in the tropifexor 30- (−29.9 U l^−1^), 60- (−34.2 U l^−1^) and 90-μg (−45.7 U l^−1^) groups versus placebo (−5.0 U l^−1^) (Extended Data Fig. 4a). At week 48, a decrease in LS mean GGT levels in both the tropifexor 140- (−35.2 U l^−1^) and 200-μg (−29.9 U l^−1^) groups was noted versus an increase in the placebo group (9.0 U l^−1^) (Extended Data Fig. 4b). Pairwise differences with 95% CIs for GGT are summarized in Extended Data Table 3. A dose-related increase in alkaline phosphatase (ALP) was observed in all parts of the study (Extended Data Fig. 4c,d).

There was no meaningful change in liver stiffness by Fibroscan from baseline to end-of-treatment in any tropifexor dose group compared with placebo. A notable change in enhanced liver fibrosis panel score was observed from baseline to end-of-treatment in the tropifexor 60-μg group (−0.25 versus +0.12 (placebo)) and tropifexor 140- and 200-μg groups only (−0.28 and −0.23, respectively, versus −0.07 (placebo)). Decreases in mean fibrosis biomarker test score from baseline to end-of-treatment in tropifexor 10–90-μg groups were not notably different compared with placebo, and neither were the LS mean decreases observed in the tropifexor 140- and 200-μg groups (data not shown).

### Patient-reported outcomes

Mean visual analog scale (VAS) itch at baseline was 1.1, 0.5, 0.7 and 0.6 for tropifexor 10, 30, 60 and 90 μg, respectively, versus 0.4 in the placebo group (Parts A + B), and 1.1 and 0.7 for tropifexor 140 and 200 μg, respectively, versus 0.9 in the placebo group (Part C). No notable differences in LS mean worsening in VAS for itch from baseline to weeks 6 or 12 were noted in the placebo or tropifexor 10–90-μg groups. Although the LS mean worsening in VAS for itch from baseline was notably greater for the tropifexor 140- and 200-μg groups versus placebo at weeks 6 (1.0 and 1.0 versus −0.1; *P* < 0.01 for both comparisons) and 12 (1.2 and 1.3 versus 0.0; *P* < 0.05 for both comparisons), the between-group differences were lost at weeks 24 (0.8 and 1.0 versus 0.5) and 48 (0.6 and 1.1 versus 0.3).

The impact on VAS for sleep disturbance due to itch was comparable between the placebo and tropifexor 10–200-μg groups up to end-of-treatment. The LS mean worsening from baseline was 0.3 and 0.5 versus 0.1 in the tropifexor 140- (*P* = 0.755) and 200-μg (*P* = 0.382) versus placebo groups, respectively, at week 48.

### Liver histology

**Extended Data Fig. 5:**
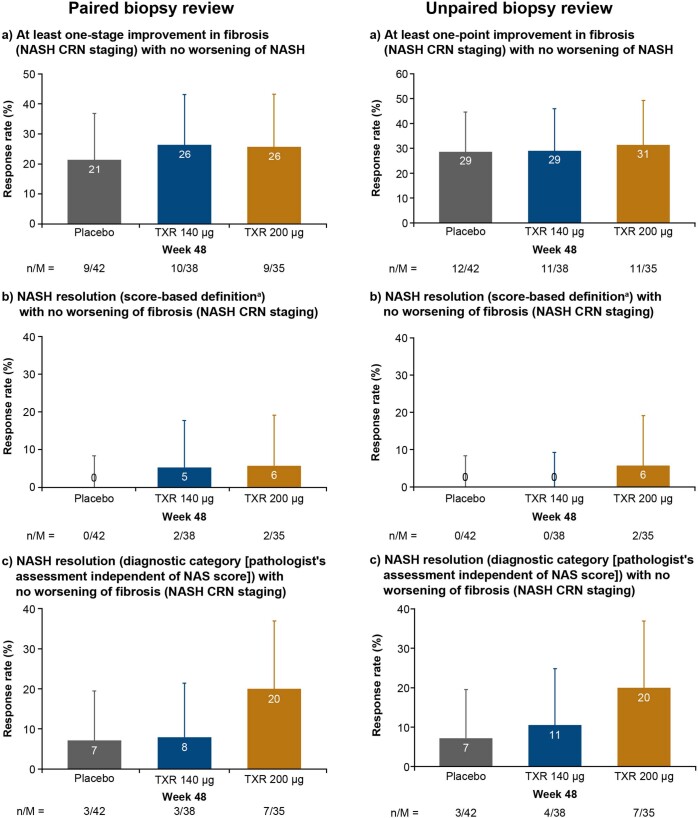
Changes in histologic features (paired and unpaired biopsy evaluation) at week 48 (Part C). a, At least one-point improvement in fibrosis (NASH CRN staging) with no worsening of NASH. b, NASH resolution (score-based definition^a^) with no worsening of fibrosis (NASH CRN staging). c, NASH resolution (diagnostic category [pathologist’s assessment independent of NAS score]) with no worsening of fibrosis (NASH CRN staging). Data are presented as response rate with 95% CI. ^a^Lobular inflammation ≤1 AND hepatocyte ballooning=0 AND any value for steatosis (definition as per FDA/EMA). CI, confidence interval; CRN, clinical research network; M, total number of subjects in the treatment group with response variable defined; n, number of subjects who responded NAFLD, non-alcoholic fatty liver disease; NAS, NAFLD activity score; NASH, non-alcoholic steatohepatitis; PBO, placebo; TXR, tropifexor.

**Extended Data Fig. 6:**
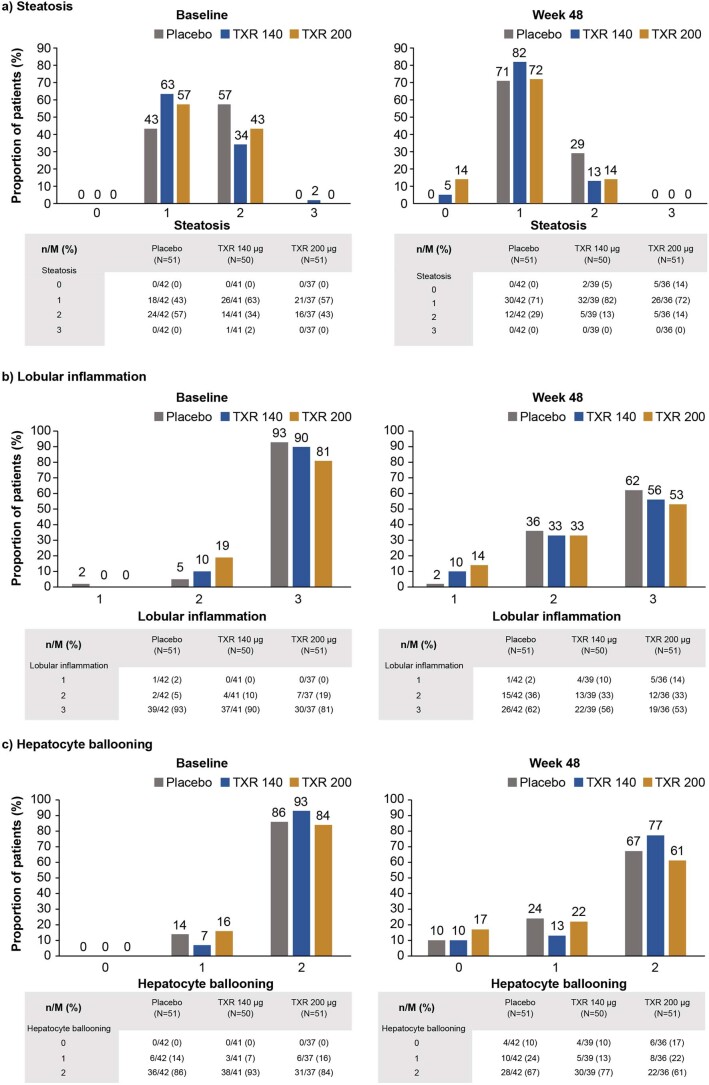
Proportion of patients in each NAS sub-score category at baseline and week 48 (paired biopsy evaluation). M, the total number of subjects with a value for a specific categorical variable; n, number of subjects who are at the corresponding category; N, the total number of subjects in the treatment group; NAFLD, non-alcoholic fatty liver disease; NAS, NAFLD activity score; TXR, tropifexor.

The results of unpaired and paired (post hoc analysis) review of biopsies are presented in Extended Data Fig. 5. The results show that the histopathology review of liver biopsies was consistent between the two modes (unpaired or paired) of evaluation. There were no notable differences among the treatment groups at week 48 in the proportion of patients who achieved ≥1 stage improvement in fibrosis (NASH clinical research network (CRN) staging) without worsening of NASH in the placebo (21% (9 of 42)) and tropifexor 140- (26% (10 of 38)) and 200-µg (26% (9 of 35)) groups (Extended Data Fig. 5a, paired biopsy review). Resolution of NASH (score-based definition: Food and Drug Administration/European Medicines Agency: FDA/EMA) without worsening of fibrosis (NASH CRN staging) at week 48 was seen in a few patients in the tropifexor 140- (5% (2 of 38)) and 200-µg (6% (2 of 35)) groups versus none in the placebo (0% (0 of 42)) group (Extended Data Fig. 5b, paired biopsy review). The central pathologist’s assessment of NASH resolution (diagnostic category: pathologist’s determination of the presence or absence of steatohepatitis) with no worsening of fibrosis was seen in 3 patients in the placebo (7%) and tropifexor 140-µg (8%) groups and 7 patients (20%) in the tropifexor 200-µg group (Extended Data Fig. 5c, paired biopsy review). At week 48, the decrease in mean total NAS was −0.9, −1.0 and −1.2 in the placebo, tropifexor 140-µg and tropifexor 200-µg groups, respectively. The proportions of patients in each sub-score category for steatosis, lobular inflammation and hepatocyte ballooning at baseline and week 48 are summarized in Extended Data Fig. 6.

### Post hoc analysis: qFibrosis and qSteatosis

**Extended Data Fig. 7:**
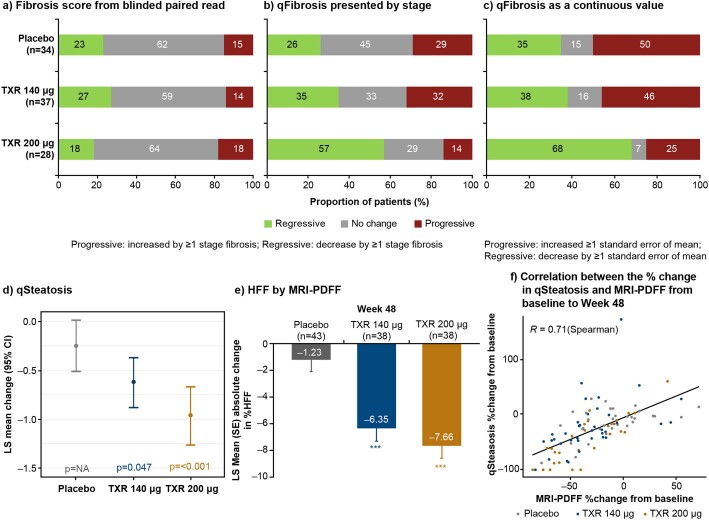
Comparison of fibrosis changes (Figures 6a-6c) and liver fat reduction (Figures 6e-6f) from baseline to week 48 (Part C). a, Fibrosis score from blinded paired read. b, qFibrosis presented by stage. c, qFibrosis as a continuous value. d, qSteatosis. e, HFF by MRI-PDFF. f, Correlation between the % change in qSteatosis and MRI-PDFF from baseline to week 48. P values for qSteatosis (figure 6d): versus placebo, from ANCOVA adjusted by baseline value, no multiplicity correction; P values for HFF (figure 6e) ***P<0.001 repeated measures ANCOVA model. ANCOVA, analysis of covariance; HFF, hepatic fat fraction; MRI-PDFF, magnetic resonance imaging-proton density fat fraction; TXR, tropifexor.

In this exploratory post hoc analysis, based on conventional scoring (CRN) of liver fibrosis, as well as by digital quantification (q) of fibrosis (qFibrosis) assessments by fibrosis stage and as a continuous value (as described in the Methods), patients were categorized as progressive, no change or regressive (Extended Data Fig. 7a–c). CRN scoring (Extended Data Fig. 7a) showed that, while a proportion of patients achieved fibrosis regression (placebo, 23%; tropifexor 140 µg, 27%; tropifexor 200 µg, 18%), the majority showed no change. In contrast, qFibrosis showed a dose-dependent increase in the proportion of patients who achieved fibrosis regression, with a marked reduction in the no-change subgroup. qFibrosis by stage (Extended Data Fig. 7b) revealed that 35% and 57% of patients in the tropifexor 140- and 200-µg groups, respectively, achieved ≥1 stage reduction in fibrosis compared with 26% in the placebo group. Similarly, qFibrosis as a continuous value (Extended Data Fig. 7c) showed a higher proportion of tropifexor-treated patients (tropifexor 140 µg, 38%; tropifexor 200 µg, 68%) achieving fibrosis reduction versus placebo (35%).

Tropifexor treatment was also associated with a dose-dependent reduction in qSteatosis (LS mean change: tropifexor 140 µg, −0.6 (*P* = 0.047); tropifexor 200 µg, −0.95 (*P* < 0.001) versus placebo, −0.25) (Extended Data Fig. 7d), consistent with HFF reduction by magnetic resonance imaging (MRI; Extended Data Fig. 7e). At the individual level, a good correlation (*R* = 0.71) was observed between changes in qSteatosis by digital quantitation and reduction in HFF by magnetic resonance imaging-proton density fat fraction (MRI-PDFF) at week 48 (Extended Data Fig. 7f).

## Discussion

In this phase 2, randomized, multicenter, double-blind, three-part study, pruritus was the most commonly reported AE with tropifexor, with an incidence that was dose-dependent. Events were generally of mild severity and led to low treatment discontinuation rates. Pruritus has been noted in other FXR studies^16,17,24^, which is consistent with a class effect of FXR agonism. Treatment with tropifexor resulted in numerically dose-dependent reductions in ALT and liver fat content (HFF measured by MRI-PDFF) at week 12 compared with placebo. Similar improvement in AST with tropifexor at week 12 was not observed.

Assessment of the secondary objectives of the study revealed that these reductions in ALT and HFF were sustained up to 48 weeks of treatment with tropifexor. Sustained reductions in GGT were also observed. Results with AST remained inconclusive up to week 48. In addition, a greater proportion of patients attained a ≥30% relative reduction in HFF at week 48 with tropifexor versus placebo. A ≥30% relative reduction in HFF has been associated with histologic improvements in NASH in several trials and has been proposed as a potential future surrogate marker for evaluating therapeutic effect in early NASH trials instead of invasive biopsies^25,26^. Patients treated with tropifexor also experienced greater weight loss after 48 weeks of treatment versus placebo, which has also been associated with histologic improvement^3,4^. Changes in lipid parameters were also observed with tropifexor, with an early increase in LDL-C and decrease in HDL-C levels, which stabilized after week 12. Although statin use was not mandatory per protocol and few investigators initiated statin treatment during the trial, data from a trial with another FXR agonist^16^, obeticholic acid, suggest that statin use can ameliorate the LDL-C elevation caused by FXR agonism. While studies of other FXR agonists have demonstrated increased drug exposure in patients with cirrhosis^27,28^, with obeticholic acid carrying a black box safety warning^29^, a hepatic impairment trial with tropifexor^30^ has shown little drug accumulation, highlighting clinically meaningful differences in PK properties, potentially related to its non-bile-acid structure and lack of enterohepatic circulation.

Despite improvement in biomarkers of liver injury and indirect indicators of histologic improvement after 48 weeks of tropifexor treatment, greater histologic improvement of fibrosis or resolution of NASH relative to placebo was not observed, based on the central pathologist’s assessment using the traditional semiquantitative NASH CRN scoring system. In a post hoc analysis, the use of second harmonic generation/two-photon excitation fluorescence (SHG/TPEF) microscopy with artificial intelligence (AI) analyses of the same liver biopsies showed that tropifexor treatment resulted in marked liver fat reduction (qSteatosis), which correlated well with the quantitative MRI-based evaluation of fat reduction and improvement of liver fibrosis (qFibrosis). Further insights into this analysis have been recently published^31^. While still an emerging technology, SHG/TPEF microscopy with AI analyses has the potential to reveal details of NASH pathobiology and, through its continuous scale assessment, highlight subtle interval changes from response to treatment which cannot be detected using conventional microscopy and traditional histologic scoring systems based on categorical fibrosis stage^32,33^. Traditional liver histopathology assessment of fibrosis is qualitative, nonlinear between stages and requires substantial changes over a relatively short time period before a response can be detected. In the current trial, the treatment period of 48 weeks may have been too short to be compared with the 72-week treatment paradigm used in other FXR agonist trials that showed histologic improvement^16,17^. To this point, recently reported results from a separate 24-week phase 2b trial investigating an FGF19 analog in patients with NASH have also shown no significant fibrosis improvement^34^.

FLIGHT-FXR Part C study limitations include a shorter therapy duration (48 weeks) than some previous FXR trials^16,17^, histologic assessment as a secondary endpoint, no preplanning for re-reading of biopsies at the end of the study and a relatively small number of patients, which limited the power to address histologic changes. Most importantly, the failure to demonstrate the expected relative histologic changes was primarily driven by the high placebo response rates compared with other FXR trials^16,17,35^ and not by low absolute response rates in patients receiving tropifexor.

At baseline, the majority of patients had markers of progressive NASH, with stage 3 fibrosis and type 2 diabetes mellitus (T2DM). Nonetheless, tropifexor demonstrated sustained improvement in liver enzymes and HFF versus placebo, with a safety profile consistent with other FXR agonists. The histologic improvements observed with tropifexor when biopsies were assessed using AI-based digital pathology offer an interesting insight into fibrosis evolution and resolution as highly dynamic processes. The dissonance between the results from traditional histologic assessments and AI-based digital pathology may be due to the latter having a greater ability to detect fibrosis changes that are not readily apparent with traditional histological assessments, or perhaps the decreased variability when using machine-based quantitative algorithms. However, in the real world, both patients and clinicians still rely on histologic results through traditional reading and the exploratory nature of the AI-based digital pathology analysis means that the results should be interpreted with caution.

In summary, treatment with tropifexor resulted in sustained decreases in ALT and HFF versus placebo. As with other FXR agonists, dose-related pruritus was frequently observed. Results from the histologic post hoc analysis support a rationale for further exploring the anti-fibrotic effects of tropifexor, either alone or in combination with other agents. Further studies are needed to characterize treatment-related changes beyond conventional histologic assessments.

## Methods

### Study design and treatments

FLIGHT-FXR (NCT02855164) was a phase 2, randomized, double-blind, placebo-controlled, dose-finding study with an adaptive design consisting of three sequential parts (Parts A, B and C). The study was conducted between August 2016 and April 2020 at 84 centers in 17 countries (Argentina, Australia, Austria, Belgium, Canada, France, Germany, India, Italy, Japan, Republic of Korea, the Netherlands, Singapore, Slovakia, Spain, Taiwan and the United States).

Study design and number of patients per treatment group are shown in Extended Data Fig. 1 and Table 1. In Part A, 77 patients were randomized (1:1:1:1:1) to receive placebo or tropifexor (10, 30, 60 or 90 μg). After the Data Monitoring Committee (DMC) review of Part A data and recommendation on dose selection for Part B, randomization to Part B commenced and 121 patients were randomized (5:4:15) to receive placebo, tropifexor 60 μg or tropifexor 90 μg. Randomization into Part C commenced after completion of Part B randomization and 152 patients (1:1:1) received placebo, tropifexor 140 µg or tropifexor 200 µg. Study medication was administered once daily for 12 weeks in Parts A and B and for 48 weeks in Part C. All patients entered a 4-week follow-up period after receiving the last dose of study treatment.

The study protocol and all amendments were reviewed by the Independent Ethics Committee or Institutional Review Board for each center. The study was conducted according to the principles of the International Council for Harmonisation of Technical Requirements for Pharmaceuticals for Human Use (ICH) E6 Guideline for Good Clinical Practice, which have their origin in the Declaration of Helsinki. Written, informed consent was obtained from each patient at screening before any study-specific procedure was performed.

### Patient population

The study included male and female patients (≥18 yr) with elevated ALT (males ≥43 U l^−1^; females ≥28 U l^−1^), HFF ≥10% at screening (as assessed by MRI-PDFF) and body weight 40–150 kg (patients with ≥4.5 kg weight reduction within the last 6 months before screening were excluded). In Parts A and B, patients with either histologic evidence of NASH (liver biopsy obtained ≤2 yr before randomization) with fibrosis stage 1, 2 or 3 and no diagnosis of alternative chronic liver diseases or phenotypic diagnosis of NASH (elevated ALT (as specified above), T2DM or elevated glycated hemoglobin (HbA_1c_ ≥ 6.5%), and increased BMI (≥27 kg m^−2^ for non-Asian race; ≥23 kg m^−2^ for Asian race), were included. In Part C, only patients with histologic evidence of NASH (liver biopsy obtained during the screening period or within 6 months before randomization) with fibrosis stage 2 or 3 (NASH CRN), and no diagnosis of alternative chronic liver diseases, were included.

Race was self-reported by the patient and captured on the demography electronic case report form.

Key exclusion criteria were previous exposure to any FXR agonist (including tropifexor), current use or history of alcohol consumption (females >20 g d^−1^; males >30 g d^−1^) for a period of more than 3 consecutive months within 1 yr before screening, uncontrolled diabetes (HbA_1c_ ≥ 9.5% within the 60 d before enrollment), presence of cirrhosis on liver biopsy or clinical diagnosis, clinical evidence of hepatic decompensation or severe liver impairment, previous diagnosis of other forms of chronic liver disease and contraindication to MRI. Patients were also excluded if they had a history or current diagnosis of electrocardiogram abnormalities indicating safety risk or were pregnant or nursing (lactating) women. Patients were excluded if taking specific medicines unless on a stable dose (within 25% of baseline dose) for at least 1 month before randomization (Parts A and B) or at least 1 month before biopsy to screening (Part C) and expected to remain stable during the treatment period. Specific medicines included anti-diabetic medications, insulin, beta-blockers, thiazide diuretics, fibrates, statins, niacin, ezetimibe, vitamin E (if doses >200 IU d^−1^; doses >800 IU d^−1^ were prohibited), thyroid hormone, psychotropic medications, estrogen or estrogen-containing birth control.

### Study design rationale and prespecified interim analysis

Four initial tropifexor doses of 10–90 μg were assessed in Part A based on preclinical results, safety and pharmacological activity (elevation of FGF19 up to 6 h after dosing) in this first-in-human study^21^. When ≥90% of the patients in Part A completed 8 weeks of treatment, an interim analysis was performed to provide data for DMC review and recommendation of doses for Part B.

Following DMC recommendation, randomization to Part B began with the tropifexor 90-μg (found to be safe and efficacious) dose and tropifexor 60 μg (the next highest dose). A second analysis was performed after all patients in Part A completed the week 16 visit. A third analysis of complete Part A and B data (pooled) was performed when all patients randomized to Part B completed the end-of-study visit (week 16) or prematurely discontinued the study. An interim analysis of Part C data (fourth planned reporting event) was performed when all patients completed the week 12 visit (time of primary endpoint) or prematurely discontinued the study. The final data analysis was carried out when all patients in Part C completed the week 52 visit.

Part C was introduced based on DMC recommendation to pursue tropifexor doses >90 μg. Randomization into Part C began after completion of Part B randomization. An exploratory exposure–response analysis of the Part A biomarker data (ALT, AST, FGF19 and GGT) at week 8 suggested investigation of area under the curve (AUC) > 40 ng × h ml^−1^ to better define a maximum biomarker response. An exploratory population pharmacokinetic (popPK) model was built using PK concentration data of tropifexor in healthy volunteers and patients with NASH. The established popPK model was used to simulate PK exposures for tropifexor 90-, 140- and 200-μg doses and to calculate the proportion of patients achieving AUC > 40 ng × h ml^−1^. The simulation suggested that at tropifexor 90-, 140- and 200-μg doses, approximately 40%, 80% and 95% of patients, respectively, may achieve an AUC > 40 ng × h ml^−1^. Thus, tropifexor 140 (predicted mean AUC ~60 ng × h ml^−1^) and 200 μg (predicted mean AUC ~80 ng × h ml^−1^) were selected for investigation in Part C to assess the therapeutic range and to characterize dose–response.

The timepoint for week 8 interim analysis in Part A and the treatment duration (12 weeks) for Parts A and B were selected based on internal recommendations. This treatment duration was also supported by Good Laboratory Practice toxicology studies (13 weeks). Further longer-term Good Laboratory Practice toxicology studies (26 weeks in rats and 39 weeks in dogs) enabled tropifexor treatment for 48 weeks in Part C to allow for evaluation of histologic endpoints and long-term safety and efficacy.

### Randomization and masking

All eligible patients were randomized in a blinded, unbiased manner using Interactive Response Technology (IRT) to one of the treatment arms. The investigator or his/her delegate contacted the IRT after confirming eligibility. A participant randomization list was generated by the IRT using a validated system which automated the random assignment of participant numbers to randomization numbers. These randomization numbers were used to link the participant to a treatment arm and unique medication number. A separate medication list was produced using a validated system which automated the random assignment of medication numbers to packs containing the investigational drug(s).

Randomization in Parts A and B was stratified by BMI (Asian <30 kg m^−2^ or ≥30 kg m^−2^; non-Asian <35 kg m^−2^ or ≥35 kg m^−2^) at baseline. Randomization in Part B was also stratified by Japanese or non-Japanese origin to ensure all treatment groups were represented in the subset of Japanese patients. In Part C, randomization was stratified by fibrosis stage 2 or 3, presence or absence of T2DM, and by Japanese or non-Japanese origin.

In this double-blind study, patients, investigator staff, persons performing the assessments, the Novartis clinical trial team and contract research organization (CRO) associates involved with continued direct study site conduct (or delegates) remained blinded to individual treatment allocation from the time of randomization until database lock for each study part (week 16 for Parts A and B and week 52 for Part C). Randomization data were kept strictly confidential until the time of unblinding and were not accessible by anyone involved in the study except for the PK bioanalyst. The identity of treatments was concealed using study drugs that were all identical in packaging, labeling, schedule of administration, appearance, taste and odor. Additional placebo capsules were given in active treatment groups when needed to maintain blinding.

During the first interim analysis (week 8, Part A), the database was locked after ≥90% of patients completed their week 8 assessments. A Novartis pharmacometrician not involved in the clinical conduct of the study and a CRO performing the statistical analysis were unblinded to the week 8 results; this facilitated data review by the DMC. During the second (week 16, Part A) and third interim analyses (week 16, Parts A + B), Novartis and CRO associates involved in data analysis and reporting were unblinded to data. For the week 12 interim analysis of Part C data, Novartis and CRO associates involved in data management, analysis and reporting, and Novartis management, were unblinded, while Novartis and CRO associates (including field associates) involved with continued direct study site conduct, site personnel and patients remained blinded.

### Procedures and assessments

Safety assessments included monitoring of AEs and SAEs, with their severity and relationship to study drug. The Medical Dictionary for Regulatory Activities (MedDRA) v.23.0 was used for the reporting of AEs.

Serum samples for the quantification of target engagement markers FGF19 and C4 were collected predose at baseline and at week 12 in Parts A and B, and predose at baseline and at weeks 12, 24, 40 and 48 in Part C. Samples were collected predose and 4 h postdose at week 6 in all parts.

Blood samples for the assessment of liver enzymes (ALT, AST, GGT, ALP) were obtained at screening, baseline and weeks 1, 2, 4, 6, 8, 12 and 16 in all parts; and additionally at weeks 20, 24, 32, 40, 48 and 52 in Part C. Hy’s law criteria (total bilirubin levels >2× upper limit of normal and ALT >3× upper limit of normal)^36^ were used in the evaluation for drug-induced serious hepatotoxicity. Body weight was also assessed at the same timepoints as liver enzymes in Parts A, B and C. Height was assessed at screening only, and waist/hip circumference at screening and week 12 in all study parts.

Fibroscan was an optional assessment; if sites had equipment available, it was performed at baseline and at week 12 in all parts and at weeks 12, 24 and 48 in Part C. Assessments at end-of-treatment were not performed in the case of premature treatment discontinuation unless the participant had received ≥8 weeks of therapy. Enhanced liver fibrosis panel and fibrosis biomarker tests were performed at screening, baseline and week 12 in all parts, and additionally at weeks 24 and 48 in Part C.

Fasting lipids were measured at screening, baseline and weeks 2, 6, 12 and 16 in Parts A and B; and at screening, baseline and weeks 2, 6, 12, 20, 24, 40, 48 and 52 in Part C. Management of treatment-emergent dyslipidemia was not prespecified in the study protocol.

Blood collection for PK was performed at week 1 (predose and 2 h postdose) and weeks 2, 4, 6, 8 and 12 (predose) in Part A; and at week 2 (predose and 2 h postdose), week 6 (predose and 4 h postdose), and weeks 4, 8 and 12 (postdose) in Part B. In Part C, blood collection for PK was performed for predose and postdose as the last activity of the visit at weeks 12, 24 and 48, and postdose as the last activity of the visit at weeks 6 and 40.

Itch severity and impact of nocturnal itch on sleep were determined on a 10-cm VAS (score range: 0 (no itch at all/no sleep loss) to 10 (the worst imaginable itch/cannot sleep at all)). Assessments were performed at screening (for sleep only), baseline and weeks 6, 12 and 16 in Parts A and B; and at screening (for sleep only), baseline and weeks 2, 6, 12, 24, 48 and 52 in Part C.

Liver MRI scans were acquired at screening and at week 12 in Parts A and B, and at baseline and weeks 12, 24 and 48 in Part C. Week 12 assessment was not done if the participant prematurely discontinued treatment before week 8. All MRI scans were performed locally (on GE, Philips and Siemens at 1.5 T and 3 T; and Hitachi at 1.5 T, whichever was available) and were evaluated by the central MRI laboratory (BioTelemetry Research, Rochester, NY, USA), blinded to the investigator, participant and sponsor until after the completion of study or study part and database lock.

In Part C, liver biopsies were obtained for all patients at baseline and week 48. Biopsies were stained using hematoxylin and eosin and Masson trichrome stains. Biopsy sections were evaluated by the central histopathologist to confirm eligibility before randomization. Paired review of biopsies was performed after all patients’ participation was completed; baseline and week 48 biopsies of each patient were read together, at the same time, by the central histopathologist, blinded to participant identification, treatment and temporal sequence of samples (baseline or week 48). NASH features in the biopsies were graded using the semiquantitative NASH CRN Histologic Scoring System. This scoring system is composed of the NAS to evaluate the key features of NASH (steatosis, lobular inflammation and hepatocellular ballooning), and the fibrosis score to evaluate fibrosis stage^37^. NAS was used to determine worsening of steatohepatitis. Two methods, diagnostic category (pathologist’s determination of the presence or absence of steatohepatitis) and score-based definition (FDA/EMA)^38,39^, were used to determine the resolution of steatohepatitis.

In addition to the central pathologist’s assessment, unstained sections of 198 paired liver biopsies (baseline and week 48) from 99 patients (fibrosis stage 2 (*n* = 42); fibrosis stage 3 (*n* = 57)) were analyzed using an SHG/TPEF microscopy with computer-assisted analyses for quantitative assessment of steatosis (qSteatosis) and liver fibrosis (qFibrosis), blinded to type of treatment, timepoint and the central pathologist’s scoring. qFibrosis is the overall output of quantitative readout of collagen parameters on a linear scale^33^. The scanning was performed on a Genesis 200, a fully automated, stain-free multiphoton fluorescence imaging microscope with AI algorithms (HistoIndex Pte.), as described previously^33,40^.

### Prespecified study endpoints

The primary endpoints included occurrence of SAEs, AEs resulting in treatment discontinuation and/or dose reductions, AEs of special interest up to end-of-study, changes in ALT and AST from baseline to week 12, and relative change in % HFF from baseline to week 12. Secondary endpoints included changes from baseline to week 12 in body weight, FGF19 and C4 levels, GGT and fasting lipid profile. Occurrence of potential itch was also assessed using VAS as a patient-reported outcome. VAS for sleep disturbance due to nocturnal itch was assessed as an exploratory endpoint. Additional secondary endpoints for Part C included the proportion of patients achieving ≥1 stage improvement in fibrosis (NASH CRN) without worsening of steatohepatitis or resolution of steatohepatitis without worsening of fibrosis at week 48 compared with baseline, changes in ALT and AST levels from baseline to week 48 and relative change in % HFF from baseline to week 48. Exploratory endpoints at week 48 included changes in total NAS and individual components.

### Post hoc analyses

Post hoc analyses included (1) assessment of histologic endpoints based on paired (baseline and week 48) review of biopsies, (2) AI-based digital quantitation of steatosis and liver fibrosis (qSteatosis and qFibrosis, respectively) in paired liver biopsies and (3) response rates at week 48 for relative HFF reduction by ≥30%. For analyzing the changes of liver fibrosis from baseline to week 48, based on the results from the paired reading by the central pathologist and from the AI-based digital quantitation (qFibrosis), patients in the placebo and both tropifexor arms were categorized as Progressor, No Change or Regressor (P/N/R analysis). The qFibrosis results were expressed both on a linear scale and by stage (F0 to F4) using an algorithm based on the blinded scoring of paired biopsies by the pathologist. For the conventional CRN scoring and for qFibrosis by stage, Progression was defined as fibrosis increase by ≥1 stage from baseline to week 48 and Regression was defined as fibrosis decrease by ≥1 stage. For qFibrosis on a linear scale, Progression was defined by increase ≥1 s.e.m. and Regression was defined as decrease of ≥1 s.e.m., based on the qFibrosis algorithm. The s.e.m. was determined when developing the qFibrosis algorithm using a cohort of 200 patients with the full spectrum of NAFLD, which included 42 patients with F2 and 57 patients with F3 stage of fibrosis. The s.e.m. for each fibrosis stage, as determined from the algorithm development, was then applied as a predetermined cut-off in qFibrosis assessment on a continuous scale in all subsequent studies (including the present one). The s.e.m. numerical values for F2 and F3 were 0.09 and 0.086, respectively.

### Statistical analysis

All participants who received at least one dose of study drug and had at least one postbaseline safety assessment were included in the safety analysis set for the assessment of safety variables. The full analysis set was defined as all participants to whom study treatment had been assigned at randomization and was used for summarizing demographic and baseline characteristics and assessment of efficacy variables. The end-of-study analysis was conducted on all participant data collected up to the end-of-study visit or the premature treatment discontinuation visit.

Analyses were performed using SAS or R programming language. The primary variables were assessed using descriptive statistics (incidence of AEs and SAEs, overall and by preferred term) and baseline-adjusted mean estimates and pairwise differences with a 95% CI from a repeated measures (in the case of multiple assessments) analysis of covariance (ANCOVA) model (ALT, AST and relative change in % HFF). All LS means are reported by treatment arm and interpretation of the comparison does not include the 95% CI (of the difference) or *P* value. ANCOVA models included the baseline assessment and treatment as covariates. Repeated measures ANCOVA also included time (visit) and interaction terms of time with baseline assessment and treatment. Baseline assessment, geographical region and BMI group (stratification factor) were included as covariates.

Missing data for ALT and AST were accounted for by using repeated measures ANCOVA (mixed-effects model repeated measures; MMRM), assuming data were missing at random. In the case of dose reduction or treatment discontinuation, any ALT or AST assessments were set to ‘missing’ for all primary efficacy analyses. Missing data for % HFF were imputed using the baseline value for the week 12 analysis. No imputation was applied for the final analysis in Part C, where an MMRM model was used. In the case of treatment discontinuation, HFF assessments obtained >4 weeks after last treatment were set to ‘missing.’

Analyses of secondary variables were also based on descriptive statistics, including change from baseline and pairwise differences versus placebo with 95% CI from repeated measures ANCOVA or pairwise ratio versus placebo with 95% CI from ANCOVA (ratio postdose versus predose for FGF19 and ratio postdose versus baseline for C4 at week 6 back-transformed from log scale). All LS means are reported by treatment arm and interpretation of the comparison does not include the 95% CI or *P* value. Binary biopsy-based endpoints were analyzed using logistic regression, including baseline fibrosis stage and BMI stratification group as covariates. Missing data for the efficacy variables were accounted for by using repeated measures ANCOVA (MMRM), as applicable, assuming data were missing at random. The same statistical methods were used for the paired review of biopsies, and only patients who had both a baseline and an end-of-treatment biopsy were included.

All *P* values shown are unadjusted for multiple testing and are therefore descriptive alone.

The primary objective of the study was to determine a safe dose or dose range. However, the assessment was to be made based on the whole safety profile and not on quantitatively formulated hypotheses for distinct parameters. Therefore, sample size was based on practicability with respect to expected speed of enrollment and duration of the study, and not on formal statistical criteria. The power considerations for efficacy assessment were based on the mean decrease from baseline in ALT seen with obeticholic acid versus placebo at week 12 (−28 (with an s.d. of 48) versus −11 (with an s.d. of 33), respectively)^17^. With sample sizes of 90 (Parts A + B) and 50 (Part C) in the tropifexor groups, and 40 (Parts A + B) and 50 (Part C) in the placebo group, the power for a *t*-test to compare both groups (one-sided type I error 0.05) would be 81% for Parts A + B and 78% for Part C.

### Reporting summary

Further information on research design is available in the Nature Portfolio Reporting Summary linked to this article.

## Online content

Any methods, additional references, Nature Portfolio reporting summaries, source data, extended data, supplementary information, acknowledgements, peer review information; details of author contributions and competing interests; and statements of data and code availability are available at 10.1038/s41591-022-02200-8.

### Supplementary information

Reporting Summary

## Figures and Tables

**Extended Data Table 1:**
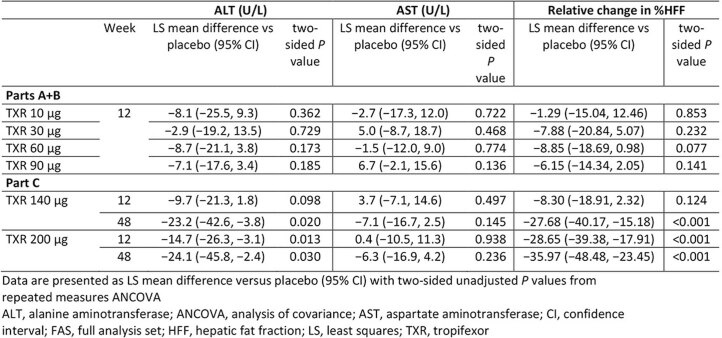
Change in ALT, AST and HFF from baseline to end-of-treatment (FAS)

**Extended Data Table 2:**
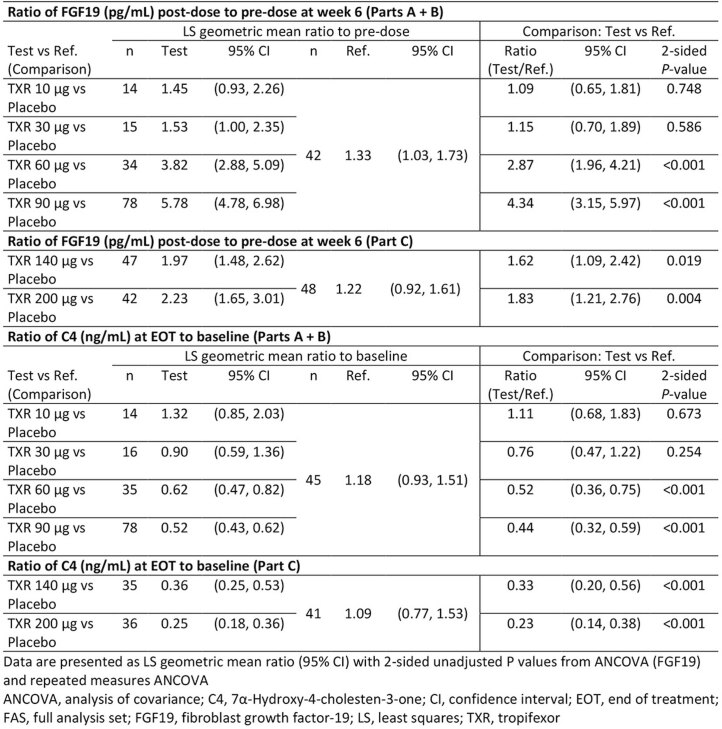
Change in FGF19 and C4 levels (FAS)

**Extended Data Table 3:**
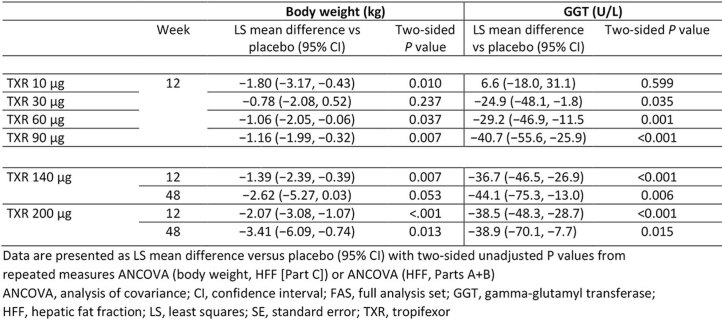
Change from baseline in body weight and GGT (FAS)

## Data Availability

The authors declare that all data supporting the findings of this analysis are available within the article and its Supplementary Information. Requests for access to aggregate data and supporting clinical documents will be reviewed and approved by an independent review panel on the basis of scientific merit. All data provided are anonymized to respect the privacy of patients who have participated in the trial, in line with applicable laws and regulations. Availability of trial data is according to the criteria and process described at www.clinicalstudydatarequest.com.
