# Supplementary material for: Tropifexor for nonalcoholic steatohepatitis: an adaptive, randomized, placebo-controlled phase 2a/b trial
Source: Nat Med. 2023 Feb 16;29(2):392–400. doi: 10.1038/s41591-022-02200-8 (PMC9941046; doi:10.1038/s41591-022-02200-8)
Supplement: Supplementary file 1 — Reporting Summary [file 41591_2022_2200_MOESM1_ESM.pdf]

nature research

Corresponding author(s): Arun J Sanyal

Last updated by author(s): Dec 21, 2022

Reporting Summary

Nature Research wishes to improve the reproducibility of the work that we publish. This form provides structure for consistency and transparency in reporting. For further information on Nature Research policies, see our Editorial Policies and the Editorial Policy Checklist.

Statistics

For all statistical analyses, confirm that the following items are present in the figure legend, table legend, main text, or Methods section.

n/a	Confirmed	
☐	☒	The exact sample size (*n*) for each experimental group/condition, given as a discrete number and unit of measurement
☒	☐	A statement on whether measurements were taken from distinct samples or whether the same sample was measured repeatedly
☐	☒	The statistical test(s) used AND whether they are one- or two-sided<br>*Only common tests should be described solely by name; describe more complex techniques in the Methods section.*
☐	☒	A description of all covariates tested
☐	☒	A description of any assumptions or corrections, such as tests of normality and adjustment for multiple comparisons
☐	☒	A full description of the statistical parameters including central tendency (e.g. means) or other basic estimates (e.g. regression coefficient) AND variation (e.g. standard deviation) or associated estimates of uncertainty (e.g. confidence intervals)
☐	☒	For null hypothesis testing, the test statistic (e.g. *F*, *t*, *r*) with confidence intervals, effect sizes, degrees of freedom and *P* value noted<br>*Give P values as exact values whenever suitable.*
☒	☐	For Bayesian analysis, information on the choice of priors and Markov chain Monte Carlo settings
☒	☐	For hierarchical and complex designs, identification of the appropriate level for tests and full reporting of outcomes
☒	☐	Estimates of effect sizes (e.g. Cohen's *d*, Pearson's *r*), indicating how they were calculated

Our web collection on statistics for biologists contains articles on many of the points above.

Software and code

Policy information about availability of computer code

Data collection	Oracle Clinical for clinical data.
Data analysis	All statistical analysis were performed using SAS and R

For manuscripts utilizing custom algorithms or software that are central to the research but not yet described in published literature, software must be made available to editors and reviewers. We strongly encourage code deposition in a community repository (e.g. GitHub). See the Nature Research guidelines for submitting code & software for further information.

Data

Policy information about availability of data

All manuscripts must include a data availability statement. This statement should provide the following information, where applicable:

- Accession codes, unique identifiers, or web links for publicly available datasets
- A list of figures that have associated raw data
- A description of any restrictions on data availability

The authors declare that all data supporting the findings of this analysis are available within the article and its appendix. Requests for access to aggregate data and supporting clinical documents will be reviewed and approved by an independent review panel on the basis of scientific merit. All data provided are anonymized to respect the privacy of patients who have participated in the trial, in line with applicable laws and regulations. Availability of trial data is according to the criteria and process described at www.clinicalstudydatarequest.com.

Field-specific reporting

Please select the one below that is the best fit for your research. If you are not sure, read the appropriate sections before making your selection.

☒ Life sciences ☐ Behavioural & social sciences ☐ Ecological, evolutionary & environmental sciences

For a reference copy of the document with all sections, see nature.com/documents/nr-reporting-summary-flat.pdf

Life sciences study design

All studies must disclose on these points even when the disclosure is negative.

Sample size	The primary objective of the study was to determine a safe dose or dose range. However, the assessment was to be made based on the whole safety profile and not on quantitatively formulated hypotheses for distinct parameters. Therefore, the sample size was based on practicability with respect to expected speed of enrolment and duration of the study, and not on formal statistical criteria. The power considerations for efficacy assessment were based on the mean decrease from baseline in ALT seen with obeticholic acid versus placebo at Week 12 (Neuschwander-Tetri, B.A., et al. The Lancet :385, 956-965, 2015). With a sample size of 90 (Parts A+B) and 50 (Part C) in the tropifexor group, and 40 (Parts A+B) and 50 (Part C) in the placebo group, the power for a t-test to compare two groups (1-sided type I error 0.05) would be 81% for Parts A+B and 78% for Part C.
Data exclusions	Apart from where described in the manuscript, no data were excluded.
Replication	This is a clinical study and measurements in patients of all treatment groups were not reassessed to check for replication. Details of randomization, blinding, drug administration and procedures are provided in manuscript to facilitate replication of outcome measures.
Randomization	FLIGHT-FXR (NCT02855164) was a phase 2 randomized, double-blind, placebo-controlled, dose-finding study with an adaptive design.<br><br>All eligible patients were randomized in a blinded, unbiased manner via Interactive Response Technology (IRT) to one of the treatment arms. The investigator or his/her delegate contacted the IRT after confirming eligibility. A subject randomization list was generated by IRT using a validated system that automated the random assignment of subject numbers to randomization numbers. These randomization numbers were used to link the subject to a treatment arm and unique medication number. A separate medication list was produced using a validated system that automated the random assignment of medication numbers to packs containing the investigational drug(s).<br><br>In Part A, 77 patients were randomized (1:1:1:1:1) to receive placebo or tropifexor (10 µg, 30 µg, 60 µg or 90 µg). After the Data Monitoring Committee (DMC) review of Part A data, and recommendation on dose selection for Part B, randomization to Part B commenced and 121 patients were randomized (5:4:15) to receive placebo, tropifexor 60 µg or tropifexor 90 µg, respectively. Randomization into Part C commenced after completion of Part B randomization and 152 patients were randomized (1:1:1) to receive placebo, tropifexor 140 µg or tropifexor 200 µg. Study medication was administered once daily for 12 weeks in Parts A and B, and for 48 weeks in Part C. All patients entered a 4-week follow-up period after receiving the last dose of study treatment.<br><br>Randomization in Parts A and B was stratified by Body Mass Index (BMI; Asian <30 kg/m2 or ≥30 kg/m2; non-Asian <35 kg/m2 or ≥35 kg/m2) at baseline. Randomization in Part B was also stratified by Japanese or non-Japanese origin to ensure all treatment groups were represented in the subset of Japanese patients. In Part C, randomization was stratified by fibrosis stage 2 or 3, presence or absence of Type 2 diabetes mellitus and by Japanese or non-Japanese origin.
Blinding	In this double-blind study, patients, investigator staff, persons performing the assessments, Novartis clinical trial team and contract research organization (CRO) associates involved with continued direct study site conduct (or delegates), remained blinded to individual treatment allocation from the time of randomization until database lock for each study part (Week 16 for Parts A and B and Week 52 for Part C). Randomization data were kept strictly confidential until the time of unblinding and were not accessible by anyone else involved in the study except for the PK bioanalyst. The identity of the treatments was concealed using study drugs that were all identical in packaging, labeling, schedule of administration, appearance, taste, and odor. Additional placebo capsules were given in active treatment groups when needed to maintain the blinding.

Reporting for specific materials, systems and methods

We require information from authors about some types of materials, experimental systems and methods used in many studies. Here, indicate whether each material, system or method listed is relevant to your study. If you are not sure if a list item applies to your research, read the appropriate section before selecting a response.

Materials & experimental systems

n/a	Involved in the study
☒	☐ Antibodies
☒	☐ Eukaryotic cell lines
☒	☐ Palaeontology and archaeology
☒	☐ Animals and other organisms
☐	☒ Human research participants
☐	☒ Clinical data
☒	☐ Dual use research of concern

Methods

n/a	Involved in the study
☒	☐ ChIP-seq
☒	☐ Flow cytometry
☒	☐ MRI-based neuroimaging

Human research participants

Policy information about studies involving human research participants

Population characteristics

The study included male and female patients (≥18 years) with elevated alanine aminotransferase (ALT; males ≥43 U/L; females ≥28 U/L), hepatic fat fraction (HFF) ≥10% at screening (as assessed by magnetic resonance imaging-proton density fat fraction [MRI-PDFF]), and body weight 40–150 kg (patients with ≥4.5 kg weight reduction within the last 6 months prior to screening were excluded). In Parts A and B, patients with either histologic evidence of NASH (liver biopsy obtained ≤2 years before randomization) with fibrosis stage 1, 2 or 3 and no diagnosis of alternative chronic liver diseases or phenotypic diagnosis of NASH (elevated ALT [as specified above], Type 2 diabetes mellitus or elevated HbA1c [≥6.5%] and increased body mass index (BMI; ≥27 kg/m2 for non-Asian race; ≥23 kg/m2for Asian race) were included. In Part C, only patients with histologic evidence of NASH (liver biopsy obtained during the screening period or within 6 months before randomization) with fibrosis stage 2 or 3 (NASH CRN), and no diagnosis of alternative chronic liver diseases were included.

Key exclusion criteria were previous exposure to FXR agonist (including tropifexor), current use or history of significant alcohol consumption (females >20 g/day; males >30 g/day) for a period of more than 3 consecutive months within 1 year prior to screening, uncontrolled diabetes (HbA1c ≥9.5% within 60 days prior to enrolment), presence of cirrhosis on liver biopsy or clinical diagnosis, clinical evidence of hepatic decompensation or severe liver impairment, previous diagnosis of other forms of chronic liver disease, and contraindication to MRI. Patients were also excluded if they had a history or current diagnosis of electrocardiogram (ECG) abnormalities indicating significant safety risk or were pregnant or nursing (lactating) women. Patients were excluded if taking specific medicines unless on a stable dose (within 25% of baseline dose) for at least 1 month before randomization (Parts A and B) or at least 1 month prior to biopsy through screening (Part C) and expected to remain stable during the treatment period. The specific medicines were: anti-diabetic medications, insulin, beta-blockers, thiazide diuretics, fibrates, statins, niacin, ezetimibe, vitamin E (if doses >200 IU/day; doses >800 IU/day were prohibited), thyroid hormone, psychotropic medications, estrogen or estrogen-containing birth control).

Recruitment

Potential study subjects were identified by the study investigators based on study eligibility requirements. As required by ICH E6 Guidelines, documented IRB/IEC approval/favourable opinion was obtained prior to providing any written information to subjects, including subject recruiting procedures (e.g. advertisements).

Ethics oversight

The study protocol and all amendments were reviewed by the Independent Ethics Committee or Institutional Review Board for each center. The study was conducted according to the principles of ICH E6 Guideline for Good Clinical Practice that have their origin in the Declaration of Helsinki. Written informed consent was obtained from each patient at screening before any study specific procedure was performed

Note that full information on the approval of the study protocol must also be provided in the manuscript.

Clinical data

Policy information about clinical studies

All manuscripts should comply with the ICMJE guidelines for publication of clinical research and a completed CONSORT checklist must be included with all submissions.

Clinical trial registration

NCT02855164

Study protocol

https://clinicaltrials.gov/ct2/show/NCT02855164

Data collection

The study was conducted between August 2016 (first patient first visit) and April 2020 (last patient last visit) at 84 centers in 17 countries (Argentina, Australia, Austria, Belgium, Canada, France, Germany, India, Italy, Japan, Republic of Korea, Netherlands, Singapore, Slovakia, Spain, Taiwan, USA).

Outcomes

Pre-specified study endpoints: The primary endpoints included occurrence of SAEs, AEs resulting in treatment discontinuation and/or dose reductions, and AEs of special interest up to end of study, changes in ALT and AST from baseline to Week 12, and relative change in %HFF from baseline to Week 12. Secondary endpoints included changes from baseline to Week 12 in body weight, FGF19 and C4 levels, GGT, and fasting lipid profile. Occurrence of potential itch was also assessed using VAS as a patient-reported outcome (PRO). VAS for sleep distrubance due to nocturnal itch was assessed as an exploratory endpoint. Additional secondary endpoints for Part C included proportion of patients achieving at least a 1-stage improvement in fibrosis (NASH CRN) without worsening of steatohepatitis or resolution of steatohepatitis without worsening of fibrosis at Week 48 compared to baseline, changes in ALT and AST levels from baseline to Week 48, and relative change in %HFF from baseline to Week 48. Exploratory endpoints at Week 48 included changes in total NAFLD activity score (NAS) and individual components.

Post-hoc study analyses: Post-hoc analyses included i) assessment of histologic endpoints based on paired (baseline and Week 48) review of biopsies, ii) AI-based digital quantitation of steatosis and liver fibrosis (qSteatosis and qFibrosis) in paired liver biopsies, iii) response rates at Week 48 for relative HFF reduction by ≥30%. For analyzing the dynamics of liver fibrosis from baseline to Week 48, based on the results from the paired reading by the central pathologist and from the AI digital quantitation (qFibrosis), patients in the placebo and both tropifexor arms were categorized as Progressor, No Change or Regressor. The qFibrosis results were expressed both on a linear scale, as well as by stage (F0 to F4) using an algorithm based in the blinded scoring of paired biopsies by the pathologist. Progression was defined as fibrosis increase by ≥1 stage from baseline to Week 48, based on conventional CRN scoring or with qFibrosis by stage, or increase by ≥1 standard error of mean (for qFibrosis on a linear scale). Regression was defined as fibrosis decrease ≥1 stage, based on conventional CRN scoring or with qFibrosis by stage, or decrease by ≥1 standard error of mean (for qFibrosis on a linear scale).
